# On Cold Abscesses

**Published:** 1855-09

**Authors:** 


*On Cold Abscesses.* By M. JOBART.—The "hot" abscess or phlegmon is developed with great rapidity, causes more or less serious functional disturbance, is the occasion and seat of severe pain, and is manifested by coloration of the skin. The "cold" abscess, on the contrary, is developed insidiously, and ordinarily without appreciable cause; it causes no local irritation, and there is no other local sign than tumefaction of the affected part as compared with the sound side. The patient does not complain of pain unless the abscess is placed in the vicinity of a nerve. There is abundant fluctuation, and sometimes on pressure being made there is a peculiar sensation felt like that imparted by grains of rice or crushing snow. In phlegmon the pyogenic membrane is thin and easily destroyed, while in cold abscess, and especially when this is somewhat old, it is very solid, whitish, with a simple alveolar organization. The pus secreted is not matured like that of the pus of phlegmon, but is somewhat viscous and semi-transparent, having white caseous lumps floating in it, and containing the débris of the false membrane which sometimes establish adhesions between the walls of the collection. Contrary to what occurs in phlegmon, these abscesses may long remain in contact with surrounding tissues without inducing changes in them. Seeing the insensible mode in which the pus is secreted, we might almost say that the cold abscess is the result of a sort of local apathy of the region affected. These abscesses are usually found where the lymphatic vessels and glands are in large numbers; and although they are most commonly found superficial, they yet often occur among deep-seated organs. Although it is generally the case that the individual is a scrofulous subject, it is far from being always so; but, at all events, the abscesses only are found in persons enfeebled from this or other causes.

All treatment must be directed to the modification of the characters of the pyogenic membrane; and if this be not modified or destroyed throughout its whole extent, we shall see happen what takes place in vulvar abscess when this membrane is not totally destroyed, viz. the reproduction of the abscess after apparent cure. Iodine injections have been much employed with a view of obtaining such modification and inducing the secretion of coagulable lymph, by which the walls of the abscess may become united. M. Jobert finds them succeed only when the abscess is recent and of small extent. In old and large collections the long incisions of Lisfranc are best, taking care to destroy all the membrane and preventing undue hæmorrhage by plugging the wound with amadou. When the skin is thin and diseased, and does not possess sufficient vitality to effect any union with subjacent tissues, a portion must be excised, and the wound healed by granulation.—*Med. Times and Gaz., from Gaz. des Hopitaux.* No. 57.

